# Assessment of Implicit Interests through an Unobtrusive Computer Task. Their Relations with Career Decision, Anxiety, and Personality Traits

**DOI:** 10.3390/ijerph182312366

**Published:** 2021-11-24

**Authors:** Antoni Castelló, Ramon Cladellas

**Affiliations:** Department of Basic, Developmental and Educational Psychology, Autonomous University of Barcelona, 08193 Barcelona, Spain; toni.castello@uab.cat

**Keywords:** unobtrusive measure, vocational decisions, personality traits, anxiety, indecision, implicit interests, software

## Abstract

Adolescence is a period where youngsters still do not know much about themselves. That makes some decisions, like those concerning vocational elections, a complicated issue that has important consequences for their life. The main goal of this piece of research is to measure implicit interests using a situated, unobtrusive computer tool (PrUnAs: Preferences Unobtrusive Assessment) as well as its connection with anxiety and personality traits: neuroticism, extraversion, self-efficacy, optimism, consciousness, and openness. Sample: 304 16-year-old adolescents enrolled in the last course of compulsory education. Instruments: Computer programs were used to measure implicit interests, career preferences, and to self-descript personality traits; finally, the paper-and-pencil test Stai was applied to measure anxiety. Results: Concordance between implicit interests and explicit choices was less than 50%. The software developed for assessing implicit interests not only proved to be an efficient tool to make them arise but also a good predictor of anxiety. Conclusions: Implicit interests and explicit elections are not the same. The approach from implicit preferences is an important shift in the approximation to vocational guiding and to reduce youngsters’ indecision level. Beyond vocational choice, this information may improve the short- and long-term quality of life and mental health.

## 1. Introduction

The transition from compulsory secondary education to post-compulsory education is an important step concerning youngsters’ vocational elections. This kind of vocational choice takes place in the developmental stage of adolescence, which is a particularly sensitive period for most individuals, where many dimensions of their personality, social bonds, and skills are still in the process of construction [1].

Notwithstanding, career decision making is a complex task that involves information about different specialties, academic demands, and professional options alongside with a thorough awareness of the own assets and preferences. It may also be a process slanted by the relative social prestige of some options or the opinions that close relatives or friends may have on which would be the better choice. At the time most teenagers make such decisions, knowledge about the specialties is usually rather blurry as well as the sureness about their own assets. Hence, many of them are forced to make a decision that will determine their future academic trajectory grounded on a weak information basis, something that frequently leads to an election that is less than optimal [2]. Implications of this election not only involve restricting the subsequent career options but also have an incidence on their emotional states [3,4] and set up a situation that may have a strong influence on their later success and happiness [5,6].

Research with adolescents has shown that their general life satisfaction is a relevant predictor of their outcomes in a variety of domains, including school and social relationships [7]. Those with higher life satisfaction also display greater physical health, enhanced social relationships, and strong academic engagement that leads to higher achievement [8,9,10,11]. Hence, the anxiety generated by a vocational decision making that does not involve implicit interests threatens life satisfaction and, despite of the optimality of the eventual election, disturbs attitudinal components, such as their involvement in whatever they are studying [12]. It may also endanger other dimensions of life that are central for most adolescents, like social life, devoting to these issues resources that should be applied to their academic lives. Therefore, if the election made was appropriate (i.e., matching individual assets), anxiety would reduce the involvement in studies, and non-academic issues—such as social relationships—could dramatically lessen the benefits of the chosen academical path. And even worse outcomes should be expected if the election was not adequate.

John Holland’s [13,14,15,16] theory has had a wide influence on career counselling both on the theoretical and practical grounds. It assesses individual interests and aptitudes throughout six areas: realistic, investigative, artistic, social, enterprising, and conventional (synthesized in the acronym RIASEC). A central contribution from Holland’s theory is that not only aptitudes are considered but also interests (i.e., the subjects or fields that are most motivating for each person) [17,18,19]. However, they are measured as explicit, declarative interests, while in our research, we consider implicit preferences or interests. Most questionnaires derived from Holland’s theory [20,21,22] assess such interests by means of explicit, verbal questions, thus introducing possible biases associated with conformity, acquiescence, or desirability. In our approach, interests are assessed through behaviour to avoid such biases.

Many of the existing instruments are also very explicit in their aim of choosing a job or a career, something that, consciously or not, may encourage them to skip their true preferences and to adjust their response to those things that are presumably desirable for the job or career. For instance, in a study by [23] a single, explicit item was used to measure individual interest for each specialty: “*How much interested (sic) are you in…?*” Similarly, in the research developed by [24], where individuals’ interest was reported as a predictor of scientific achievement, a single question was also used to estimate it: “*How much interested (sic) are you in science?*” Perhaps not many of those interested in science were motivated by the salaries associated with scientific professions, though that could have a higher probability in other specialties. In these cases, would the answer be a true measure of interest for the specialty? At least, not always.

The limitations of self-reports and declarative measures can be bypassed by approaches based on direct behaviour [25], particularly when choices take place. As an example of these approaches, it could be considered that students who are interested in a subject (for example, literature) will be more engaged in literature classes, will seek information related to literature subjects, will spend more hours reading or studying this subject, and will have more and more organised knowledge on this topic. Any of these behaviours can be directly measured or observed, thus providing a situated and non-obtrusive assessment of the underlying interests. Instead of appealing to declarative estimations about the interests, these approaches rely on spontaneous manifestations through direct behaviour [26].

The research presented in this article is mainly concerned with a situated, unobtrusive measurement of intrinsic interests through a computer program developed by the authors. The program can provide objective measures of interests and indecision, which are then contrasted with explicit career elections, self-description of some personality traits, and anxiety level.

### 1.1. Vocational Interests

Vocational interests are a traditional topic associated with individual differences [27] as well as a widespread means for characterising, comparing, and matching persons with academic specialties to specific workplaces [28]. Interests have been a central variable in areas of vocational choice [16], educational and vocational counselling [29], career development [30], and occupational success [31].

Interest is not a monolithic concept, and a clear distinction is made in the literature between individual interest [32] and situational interest [33]. This article will focus on individual interest, which refers to a stable type of interest, like a deep-seated interest in psychology, science, or any other field [34]. This kind of interest develops gradually and determines a disposition to engage and endure in a particular subject over time [35]. This kind of interest is expected to develop slowly and to be resilient to external manipulation (by fashion or group preferences, for instance). Therefore, interests can be represented as a disposition associated to personal preferences, and the fulfilling of such a disposition provides satisfaction to the person. When a given academic or professional field matches someone’s interests, the satisfaction felt contributes to a greater focusing and a longer persistence in the tasks, something that is associated with better outcomes. The opposite situation—when academic or professional activities do not match interests—acts in a negative manner, thus reducing focus and perseverance.

Moreover, scientific literature provides evidence that intrinsic compared to extrinsic interests predicts higher life satisfaction, a greater self-esteem, and more self-actualization; they attenuate depression and anxiety and provide an increase in cooperative behaviour [36,37,38,39,40,41]. Intrinsic motivation is known to guide choices when goals have a personal, internalized significance for an individual.

According to some authors, intrinsic interests and career decision could be linked [42] in the sense that a differentiated and consistent interests’ profile should facilitate the making of a career choice [43,44]. These authors [45] reported several studies conducted on high school and college students where the awareness of true interests was determinant in the career decision-making process.

The motivational effects of interests operate alongside with external motivational factors, like prestige, incomes, or satisfying the expectations of other people (e.g., parents’ expectations). Although these external factors can have powerful effects on decision making, they do not steadily improve the conditions associated with academic or work performance. It is a typical situation that external motivations cease having an effect once they have been satisfied [46]. For instance, the efforts devoted to achieving a bonus income do not generalise to the normal work situations. Indeed, they can even have a negative effect on ordinary work performance, inspiring involvement and tough work only for bonus-rewarded tasks. Most of these external motivations are goal oriented, while fulfilling the intrinsic interests is task-oriented and, to some extent, independent from the results achieved. In other words, intrinsically interested people enjoy doing the tasks and enjoy getting good outcomes, too. Externally motivated people enjoy only getting outcomes [47]. Hence, intrinsic interests permit enjoyment on a daily basis, while extrinsic interests provide specific-point enjoyment (e.g., when the salary is paid, when an award is gained, when a grade has been achieved) sometimes attached to daily unpleasant activities [48].

Despite the known advantages of intrinsic interests, they are not widely divulged. When making decisions that concern vocational choices, many people may place intrinsic and extrinsic interests in a similar level or may not consider intrinsic interest at all [49]. For instance, someone may feel that two different specialties can be equally interesting: one of them because they like very much the object of the specialty and the other one because it provides larger average salaries. In cases like this, wise counselling should weight differently the options and make clear the indirect advantages of intrinsic interests, thus resolving the apparent equality of the two options. In this sense, there is also a common misunderstanding that even when a profession has an average income lower than another profession, usually the best professionals of both fields are paid far beyond the average values. Being one of the best professionals has a great deal to do with the involvement in the task, including attitudes like orientation to constant personal improvement and love for the quality of the outcomes. These conditions will seldom be met by externally oriented people due to their orientation to the goal instead of the process [50].

### 1.2. Career Indecision

Career indecision is a construct that refers to the emergence of problems during the career decision-making process [51,52,53]. Indecision explains why some individuals are uncertain about their educational and vocational future, while others are more confident in making their choices [54]. A higher confidence in the choice does not necessarily entail that the choice is correct. For example, someone who has only compared average salaries may be quite confident about their choice if it consists of the profession that, in average, is best paid. Despite their feeling of confidence, when this single criterium is applied, they could choose a specialty that does not match neither their assets nor their intrinsic interests.

Indecision is generally regarded as a normal stage through which almost everybody passes along their lifetime [53,55,56,57], and whenever it happens, it increases anxiety, at least temporarily. In contrast to indecision, indecisiveness refers to a chronic inability to make decisions in most contexts and situations [58]. It is therefore important to distinguish between vocational indecision, which refers to a normal phase of life in which careers are chosen and pursued, and indecisiveness, which is a personality characteristic manifested by the difficulty in making decisions in any dimension of life. Using a different framing, [59] distinguished between undecided individuals—those with temporary indecision and a passing inability to choose but with the potential to decide soon—and indecisive individuals, who suffer from chronic indecisiveness, attributable to high anxiety and low problem-solving abilities. Thus, indecisiveness can be defined as a chronic state that stems from pervasive emotional and personality-related difficulties [55,58,60]. Career decision difficulties have also been associated with personality traits [61], linking these traits to indecisiveness.

### 1.3. Personality Traits

The role of personality in vocational choices has long been studied (e.g., [16,62,63,64,65]). In [66], the authors showed that personality is related to the career expectations of students. The authors of [67] inspected the relation between personality and career decisiveness, detecting a relationship between some personality traits (neuroticism, extraversion, openness, consciousness, optimism, and self-efficacy) and career indecisiveness.

Neuroticism is a characteristic of individuals that have a tendency to be anxious, depressed, angry, and insecure [68]; extraversion is described as those individuals who tend to be sociable, talkative, assertive, and active [68]; openness is associated with persons who are intellectual, imaginative, sensitive, and open-minded [68]; consciousness applies to individuals who tend to be careful, thorough, responsible, organized, and scrupulous; optimism refers to a generalized expectation that good things will happen in the future [69]; and self-efficacy is defined as the level to which the individual feels confident with their ability to successfully perform tasks in the career decision making, such as gathering occupational information, selecting goals, making plans, and problem solving [70].

Extraversion and neuroticism seem to be more associated with the difficulties involving career decision making rather than consciousness or openness to experience [71,72]. Specifically, a study conducted by [61] showed that indecisiveness displayed a negative correlation with extraversion and a positive correlation with neuroticism. In a similar direction, it has been shown that both extraversion and consciousness are associated with career motivation and career success [73].

Dispositional optimism relates to the self-regulatory model of goal-seeking behaviour, which examines how outcome expectations affect goal-setting behaviour, such as those required to achieve career outcomes. In fact, several researchers have noted potential benefits of optimism in establishing career plans [74,75,76,77] as well as in later success in leadership roles [78].

Trait-anxiety has been noticed as an important precursor of career indecision [79]. The authors of [80] suggested that anxiety is closely related to career indecision, and trait-anxiety plays a more influential role in career indecision than state-anxiety. Hence, individuals with greater trait-anxiety tend to experience more difficulties in career decision making [56,58,81] and be less satisfied with their career choice [82]. In these cases, decision making is hindered by previously existing anxiety—it existed before the choice-situation, yielding to indecision, and indecision could escalate anxiety level. On the other hand, self-efficacy is regarded as one of the significant factors that influences the process of career election. Inverse associations between self-efficacy and career indecision have consistently emerged [83,84,85,86]. It should, however, be noticed that most of the elements associated to self-efficacy consist of external criteria. They are undoubtedly related to the election process but do not include true, implicit preferences. Hence, feeling capable of making a rational choice reduces indecision though it does not ensure an optimal choice.

There are plenty of benefits after making a career choice: anxiety diminishes, self-concept consolidates, and self-efficacy rises. Then, reaching a conclusion in this concern is positive in the immediate- and short-term scope. However, in the long run, reaching a conclusion is not enough—the matching of the elected career with intrinsic interests is what is going to improve quality of life, something that will seldom happen if intrinsic interests have not been considered.

Overall, the preceding literature suggests that, although other combinations with a minor weight might exist, there are four main scenarios where vocational choices take place: (1) individuals suffering chronic indecisiveness; (2) individuals that are not completely aware of their intrinsic interests, and they do not use these intrinsic preferences for career election; (3) people who are aware of their intrinsic interests but do not use much of this information for the decision-making process; and (4) individuals that are aware of their intrinsic interests and do use the information for choosing a career. The fourth scenario is the most favourable although intrinsic interests could not be properly weighted. On the other hand, the first scenario may be difficult to deal with, as indecisiveness blocks the decision-making process.

In the second and third scenarios, however, there are opportunities to make the intrinsic interests emerge and to weight them properly. The key point is awareness of both the interests themselves and of their importance. Although someone could not be aware of their interests, if they exist, they should influence feelings and behaviour. Maybe they do not know why, but they must feel more attracted to and comfortable with some subjects. Since these kinds of feelings are closer to emotional states rather than to rational awareness, the probability of considering them when taking transcendental decisions, like making a career choice, is very low. Then, if a way to make intrinsic interests emerge is found, individuals from the second and third scenarios can gain awareness of them and deeply modify their decision-making process (i.e., move to the fourth scenario). On the other hand, people who are already aware of their interests but do not involve them in the decision-making process can be taught to do it by making them aware of their importance (maybe reducing the importance of external criteria, too).

Hence, in terms of anxiety generation, the four scenarios could be sorted as follows:

(1) The first scenario should be expected to be associated to high trait-anxiety and other personality traits. The vocational election situation would probably increase state-anxiety, which would accumulate the effects of general indecisiveness and point-indecision;
(2) Individuals from the second scenario should be expected to feel some degree of stress (and consequent state-anxiety) due to not considering their intrinsic interests. They are not aware enough of them, and for whatever the reason, they only use external criteria to make their choice. Hence, some degree of indecision is expected;
(3) Individuals from the third scenario may have moderate levels of anxiety because they will probably feel that their decision-making process is sound and rational. Moreover, it includes at least some of their implicit interests. Mild indecision should be expected; and
(4) Finally, people belonging to the fourth scenario should be expected to show a low anxiety level, as they act coherently with their intrinsic interests. Hence, they would show the lowest level of indecision.

The level of anxiety, as it has been ordered in the previous list, is expected to be inversely proportional to self-confidence as well as to the time spent in exploring contents that meet the intrinsic interests (the more the anxiety, the lesser the time spent in any content). Additionally, it should be directly proportional to the number of different contents explored, which is a direct indicator of indecision (along with low exploration time).

### 1.4. Research Goals

The specific goals and hypothesis associated to them are as follows:

1. To show that intrinsic interests are not the same than explicit career choosing, at least in those cases suffering from indecisiveness (scenario 1) and those that are not fully aware of their intrinsic interests (scenarios 2 and 3). The hypothesis associated to this goal is that intrinsic interests and career choice are not expected to match in a large part of the sample. Furthermore, results are expected to permit the classification of any individual in one of the four scenarios. Such a classification would distinguish different lines of intervention aimed to the optimisation of their vocational decision-making.
2. Independently of the fields that match the intrinsic preferences, indecision can be estimated by the computer program and be compared to the personality traits declared by the participants and a measure of their anxiety (state and trait). The associated hypothesis states that the connection of indecision, personality traits, and anxiety described in the literature will be empirically replicated with the unobtrusive measure.

## 2. Materials and Methods

### 2.1. Participants

A total of 304 students participated in this study. The sample consisted of 155 men (55%) and 149 women aged 15 to 16 years. They were taking the last year (fourth) of the compulsory secondary education in the Spanish system at six concerted educational centres from Barcelona province. They were assessed in the courses from 2019–2020 and 2020–2021; the overall number of cases belonged to twelve different groups. Table 1 displays the distribution of participants.

The data analysed in the present study is just a part of that obtained in a regular vocational orientation process developed in the educational centres. All cases participated voluntarily, and their parents were fully informed and provided explicit consent to the assessment.

### 2.2. Instruments

The authors of this piece of research previously developed computerised tasks aimed to vocational assessment. Most variables were assessed through these computerised instruments alongside one paper-and-pencil measure.

#### 2.2.1. PrUnAs

**Figure 1:**
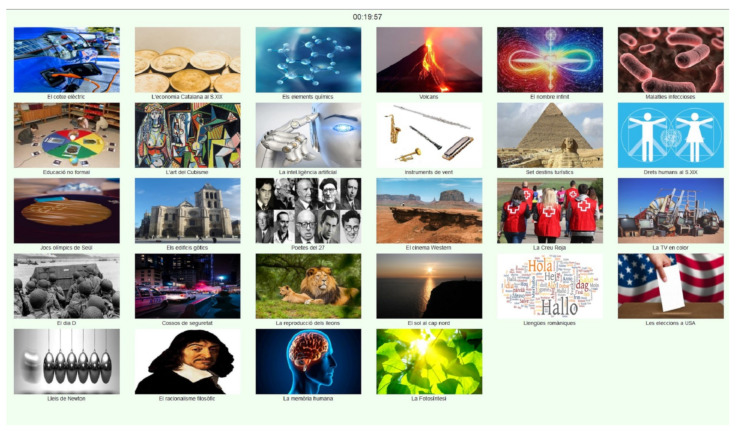
Main screen of PrUnAs application. From left to right, in rows, the titles in English are: The electric car; Catalan economy in the nineteenth century; Chemical elements; Volcanoes; The infinity; Infectious diseases; Non-formal education; Cubist art; Artificial intelligence; Wind instruments; Seven tourist destinations; Human rights in the twentieth century; Olympic games in Seoul; Gothic buildings; Poets from the 27th; Western cinema; The Red Cross; Colour TV; D-day; Security corps; Reproduction of lions; The sun at North Cape; Romanic languages; Elections at the USA; Newton’s laws; Rationalism in Philosophy; Human memory; Photosynthesis.

The computer program PrUnAs (Preferences Unobtrusive Assessment) [87] displays 28 titled images concerning different subjects, as is shown in Figure 1.

The topics represent the fields of knowledge of pure sciences, health sciences, social sciences, applied social sciences, arts and humanities, and engineering. Most of them can be linked to different majors within each field as well as to different professional-oriented studies. For instance, the topic of electric cars can be appealing both for students aimed to engineering and those that will follow professional studies on automotive mechanics or on electricity. All texts were written in Catalan, which is the vehicular language for compulsory studies in Barcelona region.

Each topic linked to a new screen where the image was enlarged, and a sentence-long description was made (15 words); a further link was provided to display a new screen where links to three different images (thumbnails) and a link to a summary (200 words) were available. Users were free to start with any of the three thumbnails or with the summary page.

**Figure 2:**
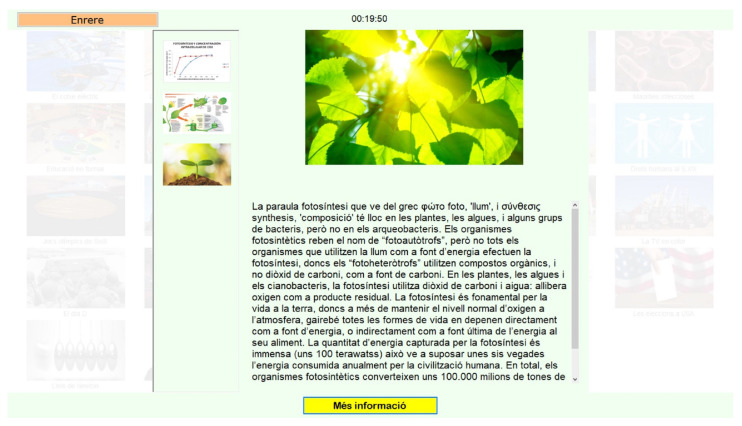
The abstract’s page. Hyperlinks to further text (yellow button) and non-textual information sources (thumbnails on the left side) are available. The top-left button is present in all pages and permits users to go back to the previously explored pages. It can also be seen that the main page is blurred underneath. The time remaining is displayed on the top. The text in English is: The word photosynthesis comes from the Greek “photo”—light—and “synthesis”—composition—and takes place in plants, algae, and some bacteria, though not in archeobacteria. The organisms that have photosynthesis are also called “photoautotrophs”. However, not all organisms that use light as a source of energy have photosynthesis, because the “photoheterotrophs” use organic compounds, instead of carbon dioxide, as the carbon source. Among plants, algae, and cyanobacteria photosynthesis uses carbon dioxide and water, and it releases oxygen as a residual. Hence, photosynthesis is central for life in the Earth, both to preserve the atmospheric normal level of oxygen, and many forms of life that depend on photosynthesis to gather energy are what many other species feed on. The amount of energy captured by photosynthesis is huge—some 100 Terawatts—approximately six times the energy consumed yearly by human civilisation. Furthermore, photosynthetic organisms fix 100,000 million tons of carbon into biomass each year. Despite it can take place in different forms, depending on the species, some of its traits are steady and regular.

On the summary page, links to a longer text (500 words) and the three former non-textual representations—an image, a concept-map, and a numerical-graphical chart—were also available. An example of this page is depicted in Figure 2.

The information was organised in hyperlinks, always providing the possibility to go back until the main page. Although it was a local, executable application (to have a strict control of time and key- or mouse-strokes) its functionality corresponded to that of a website. The participants were free to navigate within the topics or between them at their will. The overall time of the task was limited to 20 min.

#### Validation of PrUnAs

In a first stage, an expert validation was applied: 18 senior university professors—three for each main knowledge field—were asked for topics that were relevant in the knowledge field and could be attractive for 16-year-old students. They provided a list of 81 topics, and the 28 finally selected were those that satisfied the criteria of availability of information, transversality, and potential attraction for the age-scope of the study. The experts reviewed the final list of topics and agreed on the appropriateness of those of their field.

In a second stage, the program was run by a random sample of first-year students from different majors of each of the six knowledge fields. They were instructed to access whatever topic could be interesting for them, restricting time to ten minutes. The percentage of topics accessed that coincide with the knowledge field of their major was registered as well as whether the knowledge field matched one of the three topics that had more navigation time spent on them. In the following Table 2, the coincidence percentage of both values are presented alongside with the number of students that participated.

It is worth stating that the matching procedure was very strict since related fields were not accepted as a match. In that sense, there were a considerable number of pure sciences students and engineering students that selected contents of the other field. These matches were discarded notwithstanding.

Considering the strict criterium for matching and the fact that most but not all students were enrolled in the major they wanted, the matching percentages are high enough (more that 75% and more than 80%, respectively) to consider the procedure as a valid measure of implicit interests.

#### 2.2.2. Explicit Vocational Preferences

Another computer program—Careers.exe [88]—was created to select and sort by order of preference up to five majors or professional grades from a list of 120 options, which included most of the specialties available in the Spanish educational system. Participants used this software to declare their explicit intentions.

#### 2.2.3. Self-Knowledge

**Figure 3:**
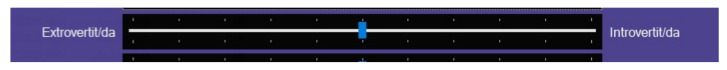
An example of a polar scale.

The application AutoDesc.exe [89] assesses twelve polar dimensions concerning personality and cognitive functioning. In this article only, the personality dimensions were considered. Each dimension placed antagonistic dimensions (e.g., detail-oriented vs. global; introvert vs. extravert) in the extremes and allowed eleven degrees of response (unnumbered), with the central one equidistant from each pole. This distribution was intended to permit an estimation of the intensity of each trait when possible but could also be converted to a ternary measure considering any response on the side of the poles as the presence of such a trait and the central point as a neutral (non-defined) response. In Figure 3, one of these dimensions (extravert vs. introvert) is displayed.

The labels of the poles were tested with a pilot group of adolescents in a Delphi approach, and many psychological terms that were meaningless for the target population were replaced by others with similar sense for them, conveyed by more common words. For the sake of illustration, originally the opposite of “detail-oriented” was “heuristic”, a term that lacked any meaning for the pilot sample and for a pilot sample of university students, too. It was replaced by “global,” which was a known word and had a similar meaning for most teenagers. To ensure that the correct meaning was conveyed by all words, the computer program displayed a “tip-tool” box with the definition when any pole was hovered on by the mouse pointer. For instance, extraversion was tipped as “those individuals who tend to be sociable, talkative, assertive, and active.” Finally, and although none of the poles could be considered as intrinsically negative, poles’ order (left and right position) was randomly determined.

The personality dimensions were derived from a set of validated and widely used personality inventories: NEO Personality inventory (PI-R), Eysenck Personality Questionnaire (EPQ), Zuckerman–Khulman Personality Questionnaire (ZKPQ), and the Big Five Questionnaire (BFQ). The personality dimensions considered in present research, according to the literature, were neuroticism, extraversion, self-efficacy, optimism, consciousness, and openness.

#### 2.2.4. STAI

Anxiety was measured with a commonly used questionnaire: The State-Trait Anxiety Inventory, STAI [90,91], in its Spanish version. This inventory was devised to assess separately the momentary, transitory anxiety (state) and the stable, enduring levels of anxiety (trait). It is composed by 40 Likert items divided in two sub-scales of 20, and each item can provide a score from 0 to 3. Hence, the maximum score for each of the sub-scales is 60. The scale has shown high reliability and validity indexes [91,92].

### 2.3. Variables

The following variables have been addressed in this study.

**Figure 4:**
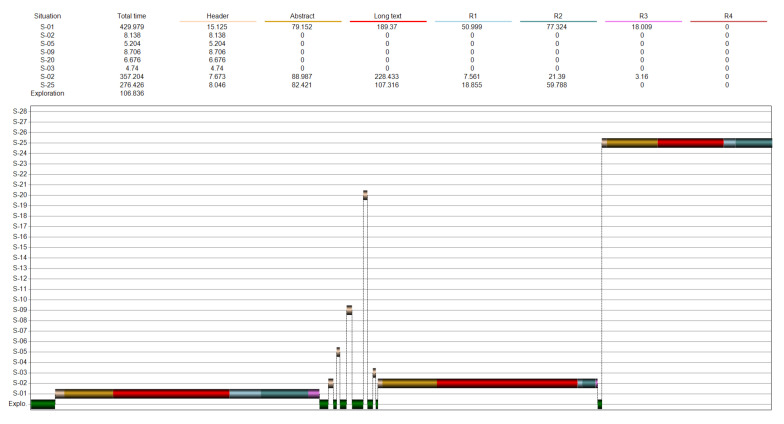
Time devoted to each of the explored topics. The colours of the bars represent the kind of information source (e.g., abstract, long-text, image, diagrams, etcetera).

*Number of situations*. The computer program registers the behaviour of each respondent, as shown in Figure 4.

Figure 4 displays the topics explored, their order, and the time spent in each topic. The green bars at the bottom of the diagram correspond to the exploration of images and titles at the main page of the program. In addition, the table on the top presents the same data numerically.

**Figure 5:**
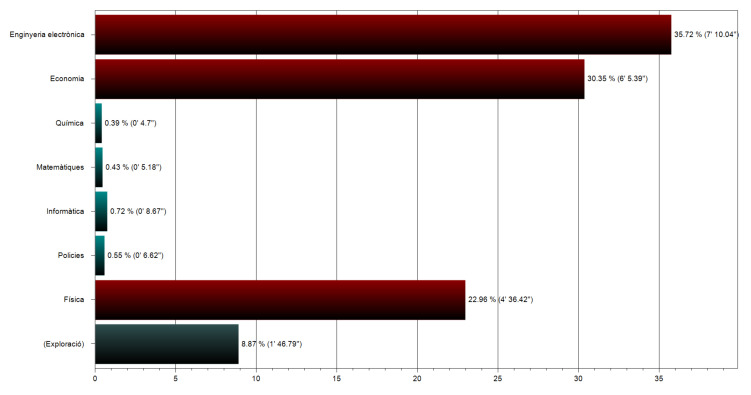
Percentage of time devoted to the accessed topics. From top to bottom, the specialities displayed are: Electronic Engineering; Economics; Chemistry; Mathematics; Computer Science; Police corps; Physics; (exploration). The percentage of time is displayed after the bars and the exact time within the parentheses.

*Situations where the respondent spent three minutes or more*. A pilot study aimed to detect how much time was needed to thoroughly read and observe the images of a topic and yielded to an average of two minutes and fifty seconds. Then, considering that the available time to do the task was 20 min, it was considered that three minutes devoted to any of the topics could be an appropriate time to consider the topic as interesting for the person. These elements were easily detected by the data compiled by the program, as Figure 5 shows.

*Explicit interests*. The studies (majors or professional grades) declared by the participants as their vocational elections.

*Coherence*. That was a computed variable that consisted of the matching of those topics where three minutes or more were spent with the first three explicit vocational options. They are indeed an operative version of the scenarios. The values that could be possible are as follows:

(1) Scenario 1. When less than three minutes were spent in any topic, since there are no intrinsic interests, the matching with explicit interests must be zero. It represents the highest level of indecision. (0 matches)
(2) Scenario 2. When three or more minutes have been spent in one or more topics, but none of these matches the explicit elections. (0 matches)
(3) Scenario 3. When three or more minutes have been spent in one or more topics, and there is a partial coincidence with the careers explicitly chosen. (1–2 matches)
(4) Scenario 4. When three or more minutes have been spent in one or more topics (a maximum of three), and there is a complete coincidence with the careers explicitly chosen. (3 matches)

Scenario 1 corresponds to the highest level of indecision, while scenario 4 represents the maximal coherence (i.e., the lowest indecision).

*Personality traits*. Personality variables are gathered through the computer program Autodesc.exe. The variables that were considered for the present study were computed in a ternary scale (left pole, neutral, right pole) since many of the observed responses were placed on the extreme values of the scale, demonstrating a rough estimation of their personality traits. The ternary score was converted to a binary variable, assigning a missing value to the neutral responses. The variables were extraversion, neuroticism (presented as stability), self-efficacy (presented as confidence), optimism, consciousness (presented as realism), and openness (presented as dynamism).

*Anxiety Stateand Anxiety Trait*. Scores obtained in STAI questionnaire.

### 2.4. Procedure

The study was introduced to the participants as a research that focused on their interests on topics that where related to academic studies as well as their way of being and behaving. Confidentiality and voluntary participation were ensured. The administration of the tasks was always supervised by the authors of this piece of work. Tasks were performed along a morning school period (from 9 a.m. to 1 p.m.) in a classroom properly prepared.

The computer programs were installed and tested in all students’ personal computers beforehand. The order of the tasks was:

(1) *Interests and preferences* (with the software PrUnAs.exe). They solved a reduced version (only four topics, different from the experimental ones, and just five minutes) of the program with a supervisor to make them familiar with the functioning of the software. After that, they ran the real version (with 28 topics) for 20 min.
(2) *Explicit election* of future studies through the software Careers.exe. It included 120 majors and professional studies. Participants could choose up to five ordered specialties. Participants had 15 min to make their choices.
(3) *Self-description* through the software Autodesc.exe. Before the real task, a supervisor exemplified how the program worked and stated that the task was aimed to assess how each of them considers that he/she is. Afterwards, they had fifteen minutes to respond. The initial state of the sliders was with all the scores in the central, neutral position. Depending on whether they considered themselves as closer to one of the poles, they could move the slider in that direction according to the intensity of the perceived trait. Twenty minutes were assigned to this task.
(4) Finally, the *STAI* questionnaire was administered. They responded in the conventional paper-and-pencil way to the Spanish version of this scale. There was no time-limit though all questionnaires were answered in less than thirty minutes.

After each task, a rest-time of five minutes was provided, and after the second task, participants could leave the classroom and take a rest on the playground for thirty minutes.

## 3. Results

### 3.1. Statistical Analysis Overview

Firstly, the number of matches between the implicit and the explicit interests was computed and transformed into a percentage. At this step, and according to the number of matches, participants were also distributed into one of four categories (scenario 1 to 4).

Secondly, a chi-squared test was performed with the four scenarios and the dichotomic personality variables.

The third step consisted of an analysis of variance (one-way ANOVA) where the independent variable was defined by the four scenarios (1 to 4), and the dependent variables were the number of explored situations, trait-anxiety, and state-anxiety.

Fourthly, a *t*-test with independent samples was run, where each explored personality trait acted as independent variable, and the former three quantitative variables were the dependent variable.

Finally, Pearson correlations among the three quantitative variables were computed.

Calculations were performed with the SPSS/PC + statistical package (version 19), and all statistical tests were bilateral with variable type I error at 5%.

### 3.2. Objective 1

The matching between those topics that received at least three minutes of participants’ attention and their respective explicit elections was of 45.2%. Consequently, 54.8% (100 − 45.2%) of the explicit elections where not congruent with the implicit preferences of the participants.

According to the four scenarios and corresponding scenarios previously described, the distribution of cases is as shown in Table 3.

### 3.3. Objective 2

#### 3.3.1. Result 1

It can be observed in Table 4 that variables self-efficacy and consciousness display statistically significant differences among the four scenarios. In both variables, the percentage of cases that perceive the trait in themselves increases with coherence, while the perception of the opposite trait decreases. Nevertheless, when analysing consciousness, it must be taken into consideration that all cases belonging to scenario 1 consider themselves as conscious.

#### 3.3.2. Result 2

The comparison of the number of topics explored within the four scenarios yielded the results presented in Table 5.

Observed differences are statistically significant (*p* < 0.001), and a trend can be detected as the means decrease as coherence increases. The average number of situations explored by participants of scenario 1 almost doubles the average number of topics explored by the members of scenario 4.

Table 6 displays the results obtained when anxiety is the dependent variable.

**Figure 6:**
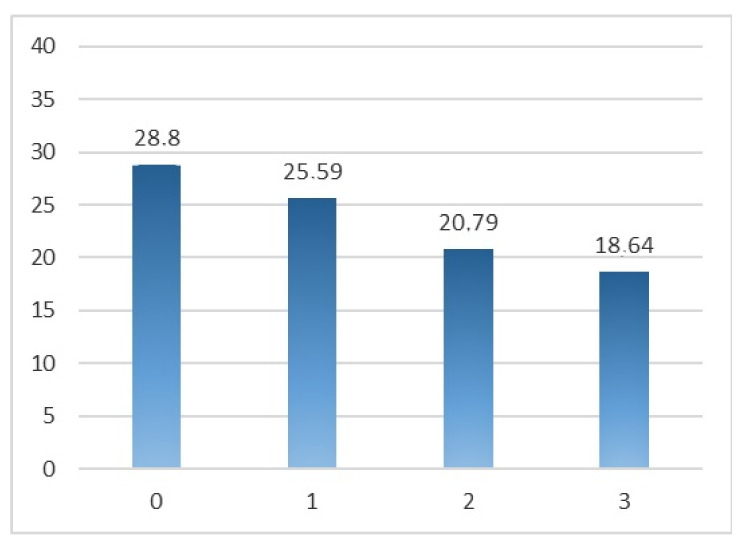
Average trait-anxiety by scenario.

**Figure 7:**
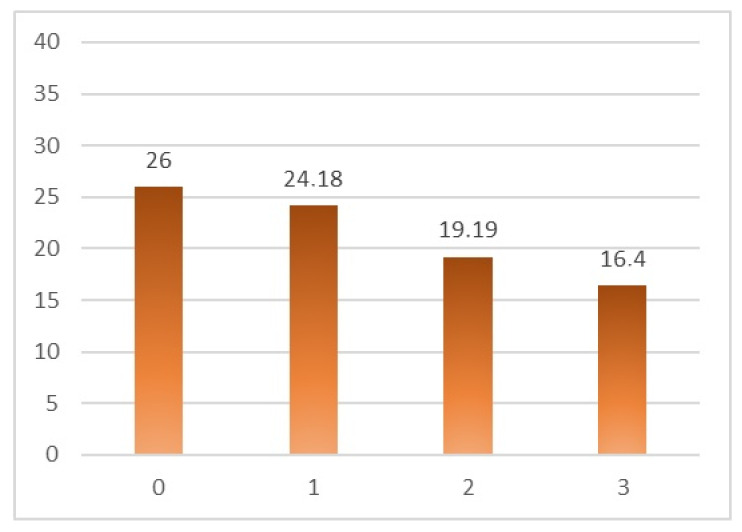
Average state-anxiety by scenario.

It can be observed that both types of anxiety decrease as congruence increases, as it is graphically presented in Figure 6 and Figure 7.

#### 3.3.3. Result 3

Significant differences can be observed for all personality traits when state-anxiety is the dependent variable. Trait-anxiety showed a similar pattern except for extraversion. Additionally, the number of topics explored showed non-significant results both for extraversion and for optimism. Hence, neuroticism seems to be associated with a larger number of topics explored, the opposite of what happens with those that see themselves as self-efficient, conscious, and open. These results are presented in Table 7.

#### 3.3.4. Result 4

Table 8 displays how the correlations are significant in all cases. It is worth noting the positive association between the number of explored topics (which is a measure of indecision/indecisiveness) and both types of anxiety.

## 4. Discussion

The main goal of this research is to measure implicit interests using a situated, unobtrusive tool. Furthermore, it is also investigated how the indexes of interest and indecision provided by the computer program are related to anxiety and personality traits.

### 4.1. Objective 1

The hypothesis associated to this goal states that intrinsic interests and career choice are not expected to match in a large part of the sample. Results show that only 45.2% of the explicit elections match with the implicit preferences of the participants. Hence, more than half of the explicit elections (54.8%) do not match the implicit interests. Furthermore, the percentage of cases with a full match between interests and explicit elections only reaches 25%, with 40% having a partial match and close to 35% having no match at all. Fortunately, the number of cases that suffer indecisiveness is moderate—just a 3.9%—but almost 31% of teenagers not suffering indecisiveness do not include their implicit interests in their explicit elections.

This lack of coherence suggests that external factors have a strong influence on explicit elections, and true preferences are not considered in many cases. Results also support that indecision is associated with the four scenarios, becoming greater as coherence becomes smaller. Scenario 1 encompasses those cases that suffer from indecisiveness and who show no particular interest for any topic. In scenario 2, people show interest for a few topics though they do not link this interest with their explicit elections. Individuals belonging to scenario 3 also have implicit interests, and some of them are considered for selecting future studies. Finally, persons from scenario 4 are aware of their interests and involve them in the election of their future educational path.

Hence, nothing opposes hypothesis number one.

### 4.2. Objective 2

The associated hypothesis states that the connection of indecision, personality traits, and anxiety described in the literature will be empirically replicated with the unobtrusive measure.

PrUnAs software allows swift and practical estimation of indecision and indecisiveness in the first case throughout the number of topics explored and in the second case by the absence of topics explored by at least three minutes. Indecision has shown significative association with some personality traits, as previously declared by [61], as well with anxiety, in concordance with research results [79,80]. Rather than a mere replication of outcomes, the use of an unobtrusive, situated measure is the novelty.

As declared by [80], anxiety is also associated with personality traits. However, extraversion showed no significant differences when anxiety-trait was considered. Neither were there significant differences when extraversion was the independent variable, and indecision was the dependent variable. However, other researchers found that extraversion, optimism, and consciousness had an inverse association with indecision, while neuroticism had a positive association [61,69,73]. Our data only confirm the positive association with neuroticism and the inverse association with consciousness. It has also been observed that there is an inverse relationship between indecision and self-efficacy, as was also detected by other studies [83,84,85,86].

Indecision can be assessed both by the scenario where participants are grouped or by the number of explored situations. Indecision is associated to a lack of confidence and consciousness.

Hence, the second hypothesis fits the results obtained with respect to anxiety, but personality traits do not completely match the hypothesized results.

### 4.3. Other Results

Results focus on implicit interests (or motivational interests), which can be considered as something different than the explicit elections. If they were the same, a much higher consistency should have been found. Then, if they are something different, traditional, declarative measures cannot be considered as a good way to appraise implicit interests.

The computer-based assessment procedure materialised in the PrUnAs software permits a correct capture of implicit interests because it almost does not require explicit decisions—participants can navigate freely, and only when something captures their interest do they spend more time accessing that information, either written or graphical. Conversely, spending more time in a few topics usually implies exploring a lesser number of situations. The title and picture of each topic that is displayed in the main page (where all topics can be accessed) is just a first contact with the subject. The architecture of the program makes the process of unveiling all the available information gradual. Hence, topics that are not attractive to a person can be easily detected at the first or second steps (brief description page and summary and thumbnails page, respectively). Those participants that go further and read all the contents or watch all the images are not forced to do so. Thus, it is reasonable to assume that their behaviour is motivated by their true interest into the topic although it is not obvious for them that they are expressing this interest behaviourally instead of declaring it. This behaviour will take place independently, so they are able or not to declare that the topic is interesting for them, as observed by [26].

The incidence of personality traits is rather more confusing since there are plenty of connections between them and anxiety. This myriad of connections suggests that there might be solid causations or interactions between personality traits and anxiety, as was found by [80]. This network of effects might mask those caused by indecision and congruence. However, strong differences have been observed as associated with these two non-personality variables.

People belonging to scenario 1 are solid candidates to suffer from indecisiveness, whatever the causes are. They certainly need psychological support to deal with a situation that may taint many dimensions of their life. In addition, members of scenario 2 and, to a lesser extent, scenario 3 show some degree of indecision, which can also be solved by psychological intervention or counselling. Detecting these people through a procedure that is not precisely aimed to this goal is an extra contribution.

However, it should not be omitted that a central value of implicit interests goes far beyond the election of a career or moderating the stress or anxiety generated by this decision. The key point is choosing a specialty that may provide daily contact with contents or topics that are attractive for the person. As was stated by [3,4,5,6], this is a great contribution to future quality of life and will enhance involvement in either studying and understanding or in professional activities. Involvement is the main factor behind success.

Despite the contributions of this piece of research, there are still many points to make clear. The relationships detected should be deepened, particularly to determine their direction and to isolate the weight of each variable. Explicit interests should also be investigated in more detail since they may include some culturally rooted arguments—maybe sophistic, at least in part—along with sound reasons. Finding the appropriate balance is an interesting goal for counsellors.

### 4.4. Limitations

As it happens in all empirical studies, the present investigation has some limitations that should be considered when interpreting the results. First, we considered a specific sample drawn from one country (Spain) and region (Barcelona area). However, as our findings are in line with many other studies, we expect the results to be generalizable to other situations. Second, respondents in this study were limited to the fourth course of secondary compulsory education. Knowing the conditions of career indecision as early as possible will be more profitable. Hence, it would be desirable to gather data from other age groups (from 14 years old on) to verify whether the groups that have been found change or not. Moreover, personality and anxiety variables are reported to display gender differences. The present research focused on general tendencies although it does not mean that gender is irrelevant. However, this issue cannot be attained in a merely descriptive approach. Deepening in the reasons of such differences was currently out of scope, but is a relevant line of future research.

## 5. Conclusions

Vocational interests have been assessed in a distinctive manner thanks to the software technology employed. It permitted to measure variables that are almost impossible to be measured by traditional, declarative approaches through a situated, unobtrusive task. Implicit preferences were supposed to be present in the declarative behaviour, mixed with other elements. However, results showed that its presence was not general. Both the methodological approach and the results constitute a relevant innovation in the field of vocational orientation and counselling.

The computer program is a necessary tool, but the design of the procedure is what is more important. Firstly, the approach is based on topics, not on grades or careers. Secondly, the overall appearance of the task is closer to an Internet portal rather than a traditional test, something that makes it an ecologically situated task for these generations that have grown up surrounded by technology. Thirdly, what is being measured as an indicator of implicit preferences and indecision (time and number of situations explored, respectively) has no intuitive access by the respondents, making it difficult—though not impossible—to be faked. Slants, like acquiescence or “good answers,” are very blurry in this kind of task. The design of gradual access to information also favours the detection of true interests since participants must explicitly click on “further information” buttons or on thumbnails and always have an easy way to go back to other topics. Hence, the normal behaviour to be expected consists of going deeper in a topic only if it is interesting for the user and to abandon those topics that inspire mild or low motivation.

Furthermore, the patterns of response also predict indecisiveness—not just indecision—which is a rather pathological state that affects many more dimensions of life than just vocational elections. Hence, this is a good chance to expose the individuals that are candidates to such a pathology and derive them to eventual therapy approaches whenever the diagnose is clinically confirmed. Anxiety is also predicted reasonably, so warnings concerning anxiety-related problems can also be raised.

However, the central contribution is that implicit interests can be measured independently of explicit elections, and they do not coincide. In this sense, vocational elections are less important than the benefits obtained when doing something that matches implicit preferences. As it has been stated, vocational decisions generate short-term well-being, and they can be modified—as a given choice does not impede changing to another career afterwards. On the other hand, studying and working on something that meets such implicit interests is a long-term investment in well-being, producing benefits in many other dimensions of life.

This is an important shift in the approach to guiding and counselling adolescents, particularly those that show difficulties in making a vocational decision. Reducing indecision and increasing the weight of implicit preferences in the “decision-making equation” raises the probability of making a good choice both in the short and long term. Indecision has immediate negative effects on happiness and general well-being, as some researchers [93] have found. Improving the quality of vocational-professional guidance also improves the emotional regulation and reduces anxiety levels in youngsters [94,95]. Previous studies have illustrated that individuals with high trait-anxiety tend to hold too much information and engage in erratic exploration behaviours and usually lack commitment to the chosen career [96,97]. Thus, it would be very relevant that counsellors encourage individuals with high trait-anxiety to more efficient and focused exploration behaviours, incorporating their preferences and not only external criteria so that they can reach a satisfactory career decision.

Gaining awareness about implicit interests and their long-term effects is a critical goal for all kind of students, even for those of scenario 4. Beyond its positive effects on vocational election, it also benefits their self-knowledge and indirectly improves their self-esteem. That will entail benefits for their mental health and well-being in the long run.

## Figures and Tables

**Table 1:** Distribution of participants by center and year.

	Year	
Center	2019	2020
	N = 140, % = 46.1	N = 164, % = 53.9
1	25	30
2	24	28
3	23	25
4	22	26
5	23	29
6	23	26

**Table 2:** Number of matches between the topics explored and current major and the topics with the longest exploration time and current major.

Knowledge Fields	Matching Explored Topics (%)	Matching Longest Explorations (%)	Number of Students	Pondered Explored	Pondered Longest
Pure Sciences	73	88	148	10.69	12.88
Health Sciences	65	72	227	14.59	16.17
Social Sciences	84	92	215	17.86	19.56
Appl. Social Sciences	81	89	232	18.59	20.42
Engineering	65	71	82	5.27	5.75
Arts and Humanities	86	91	107	9.10	9.63
Sum			1011	76.10	84.41

**Table 3:** Distribution of cases by scenario.

	Scenario				
	1	2	3	4	Total
Number of cases	12	94	122	76	304
Percentage	3.9	30.9	40.1	25.1	100

**Table 4:** Contingency table between scenarios and personality traits.

		Scenario						
Personality Trait		1 (%)	2 (%)	3 (%)	4 (%)	Total (%)	Chi-square	*p*
Extraversion	No	1.5	10.4	12.3	9	33.2	0.199	0.978 *
	Yes	2.6	20.5	26.5	17.2	66.8		
Neuroticism (stability)	No	2.9	164	13.6	10	42.9	3.920	0.270 *
	Yes	3.6	13.6	20.7	19.3	57.1		
Self-efficacy (Confidence)	No	2.9	22.4	26.4	12.6	64.3	11.568	0.009 *
	Yes	1.1	7.9	13.4	13.4	35.7		
Optimism	No	2	13.9	16.3	7.6	39.8	4.523	0.210 *
	Yes	2.4	16.3	23.1	18.3	60.2		
Consciousness (Realism)	No	0	11.5	14.1	4.8	30.4	10.221	0.017 *
	Yes	4	18.9	26	20.7	69.6		
Aperture (Dynamism)	No	1.3	14.2	14.6	10	40.2	0.969	0.809 *
	Yes	1.7	18	25.1	15.1	50.8		

Note: * *p* < 0.05

**Table 5:** Number of situations explored by scenario.

	Scenario					
	1	2	3	4	*F*	*p*
Number of situations	14.42 ± 4.74	10.83 ± 4.43	8.91 ± 3.86	7.28 ± 3.55	17.91	0.000 **

Note: ** *p <* 0.001.

**Table 6:** Anxiety score (trait and state) by scenario.

	Scenario					
	1	2	3	4	*F*	*p*
Anxiety State	26 ± 8.14	24.18 ± 11.08	19.19 ± 11.26	16.40 ± 8.60	5.76	0.001 *
Anxiety Trait	28.80 ± 6.56	25.59 ± 9.30	20.79 ± 9.71	18.64 ± 8.15	7.03	0.000 **

Note: * *p* < 0.05; ** *p <* 0.001.

**Table 7:** Number of topics explored, trait-anxiety, and state-anxiety by personality traits.

		Number of Explored Topics			Anxiety Trait			Anxiety State		
Personality Trait		Mean	*t*	*p*	Mean	*t*	*p*	Mean	*t*	*p*
Extraversion	No	9.55	−0.064	0.949	23.82	1.528	0.130	24.72	4.330	0.000 **
	Yes	9.51			21.29			17.28		
Neuroticism (stability)	No	8.15	3.253	0.001 **	19.44	4.786	0.000 **	16.29	6.115	0.000 **
	Yes	10.60			26.32			26.48		
Self-efficacy (confidence)	No	10.67	7.219	0.000 **	25.20	5.079	0.000 **	23.00	4.054	0.000 **
	Yes	7.23			17.63			15.43		
Optimism	No	9.85	1.104	0.271	29.80	9.604	0.000 **	24.69	4.321	0.000 **
	Yes	9.24			17.27			16.89		
Consciousness (realism)	No	10.55	2.978	0.003 *	25.81	3.084	0.003 *	26.90	4.842	0.000 **
	Yes	8.90			19.93			16.86		
Openness (dynamism)	No	10.24	2.249	0.025 *	24.54	3.687	0.000 **	22.54	2.087	0.039 *
	Yes	9.00			18.66			18.37		

Note: * *p <* 0.05; ** *p <* 0.01.

**Table 8:** Correlations between topics explored, state-anxiety, and trait-anxiety.

	Number of Topics	Anxiety State
Anxiety State	0.316 **	
Anxiety Trait	0.324 **	0.774 **

Note: * *p* < 0.05; ** *p* < 0.01.

## Data Availability

The data presented in this study are available on request from the corresponding author.
